# Three-year observations on the effect of different cusp inclinations on the restoration of short maxillary first molar implants: A randomized controlled trial

**DOI:** 10.3389/fphys.2022.992800

**Published:** 2023-01-04

**Authors:** Yuye Cheng, Cong Xiao, Yu Zhu, Qiuyan Chen, Longbo Zhang, Yanshu Zhang, Meiqin Gao, Xinpei Li, Yan Zhou, Guiqiang Song, Tiecheng Zhang, Runsheng Pei

**Affiliations:** ^1^ Department of Prosthodontics, Nantong Stomatological Hospital, The Affiliated Nantong Stomatological Hospital of Nantong University, Nantong, Jiangsu, China; ^2^ Department of Orthodontics, Nantong Stomatological Hospital, The Affiliated Nantong Stomatological Hospital of Nantong University, Nantong, Jiangsu, China; ^3^ Nantong Stomatological Hospital, The Affiliated Nantong Stomatological Hospital of Nantong University, Nantong, Jiangsu, China; ^4^ Department of Anesthesiology, Nantong Stomatological Hospital, The Affiliated Nantong Stomatological Hospital of Nantong University, Nantong, Jiangsu, China

**Keywords:** cusp inclination, short implant, alveolar bone height, depth of periodontal probing, implants

## Abstract

**Objective:** To investigate the effect of different cusp inclination on short implant prosthesis of maxillary first molar after 3 years of weight-bearing in biology and mechanics.

**Methods:** The clinical patients were randomly selected from the database and divided into four groups A, B, C, and D according to the cusp inclination of the maxillary first molar short implant restoration (4.8 mm × 8 mm, Dentium). 20 cases in each group. The cusp inclination was 10 degrees–15 degrees, 15 degrees–20 degrees, 20 degrees–25 degrees, 25 degrees–30 degrees. After 3 years of weight-bearing, cone beam computed tomography (CBCT) and Florida probe were used to measure and observe the height of alveolar bone (H), periodontal probing depth (PD) and modified sulcus bleeding index (MBI). Visual analogue scale (VAS) was used to evaluate the overall satisfaction of patients, and the mechanical complications of each group within 3 years of implant weight-bearing were counted.

**Results:** The H and PD of group D were 1.09 ± 0.23 and 2.19 ± 0.11 respectively, which were significantly higher than those of group A, B and C (*p* < 0.05). There was no significant difference in MBI between groups A–D (*p* > 0.05). The VAS scores of group B and group C were 88.36 ± 5.12 and 88.70 ± 4.52 respectively, which were higher than those of group A and group D (*p* < 0.05). The incidence of food impaction, porcelain collapse and abutment loosening in group D were 40.0%, 25.0% and 15.0% respectively, which were higher than those in group B and C (*p* < 0.05).

**Conclusion:** The risk of biological and mechanical complications increases after long-term weight-bearing of maxillary first molar short implant prostheses with high cusp inclination. The cusp inclination of short implant prostheses should be designed as low as 25 degrees.

## 1 Introduction

Implants are an oral surgical technique designed to replace lost teeth with fixed restorations by inserting dental implants made of titanium or other biocompatible materials into the jawbone (Glücker et al., 2020; Cutolo et al., 2022). The vertical bone augmentation and major reconstruction procedures that accompany the placement of standard-length implants often result in prolonged treatment times and increased risk of postoperative complications (Thoma et al., 2017). The length and diameter of implants have decreased significantly over the past few decades (Monje et al., 2014). The application of short implants simplifies the treatment process, shortens the treatment time for patients, reduces more invasive procedures, and expands the application range of implant dentures. Compared with standard implants, the 3-year–5-year success rate of short implants has also been confirmed in recent years (Brocard et al., 2000; Stellingsma et al., 2004; Misch et al., 2006). According to reports, after a mid-term follow-up, the use of short (length ≥ 6 mm and ≤ 8 mm) or ultrashort (length ≤ 5 mm) implants in rehabilitation of extreme maxillary and mandibular atrophy has similar effects on survival and bone-level stability compared to implants using standard length implants (Bechara et al., 2017; Altaib et al., 2019; Felice et al., 2019).

Implant stability and long-term clinical success are affected by various factors, such as implant length and diameter, design, microscopic morphology of the implant surface, implantation technique, implant shape as well as the consistency of the implant and the surrounding bone (Sodnom-Ish et al., 2020; Kim et al., 2021). How to improve the design of implant dentures to reduce the impact on implants and surrounding tissues has always been a research hotspot in the field of oral implantology (Brune et al., 2019). Studies have shown that the occlusal design, especially the cusp inclination, has a significant impact on conventional implants (Manchikalapudi and Basapogu, 2022). In the restoration of implant and maxillofacial surfaces, it should be considered that implant dentures are different from natural teeth. An effective method for controlling occlusal force is to control the cusp inclination (Kim et al., 2005). However, in the case of larger crown/implant ratios, the impact of occlusal design on soft and hard tissues around short implants is less studied, and there is still insufficient evidence for the effect of cusp inclination on short implant restorations. For patients with maxillary first molar implant restoration, does different cusp inclination have a significant effect on the amount of alveolar bone resorption at the neck of the implant restoration? Can low cusp inclination reduce the incidence of mechanical complications within 3 years of implant restoration? In this study, by observing the level of cervical bone resorption, periodontal probing and surrounding soft tissue after 3 years of short implant restorations in patients with different cusp inclination groups, the effect of different cusp inclination occlusal designs on the surrounding soft and hard tissues after 3 years of short implant restorations was investigated.

## 2 Materials and methods

### 2.1 Study design

**FIGURE 1:**
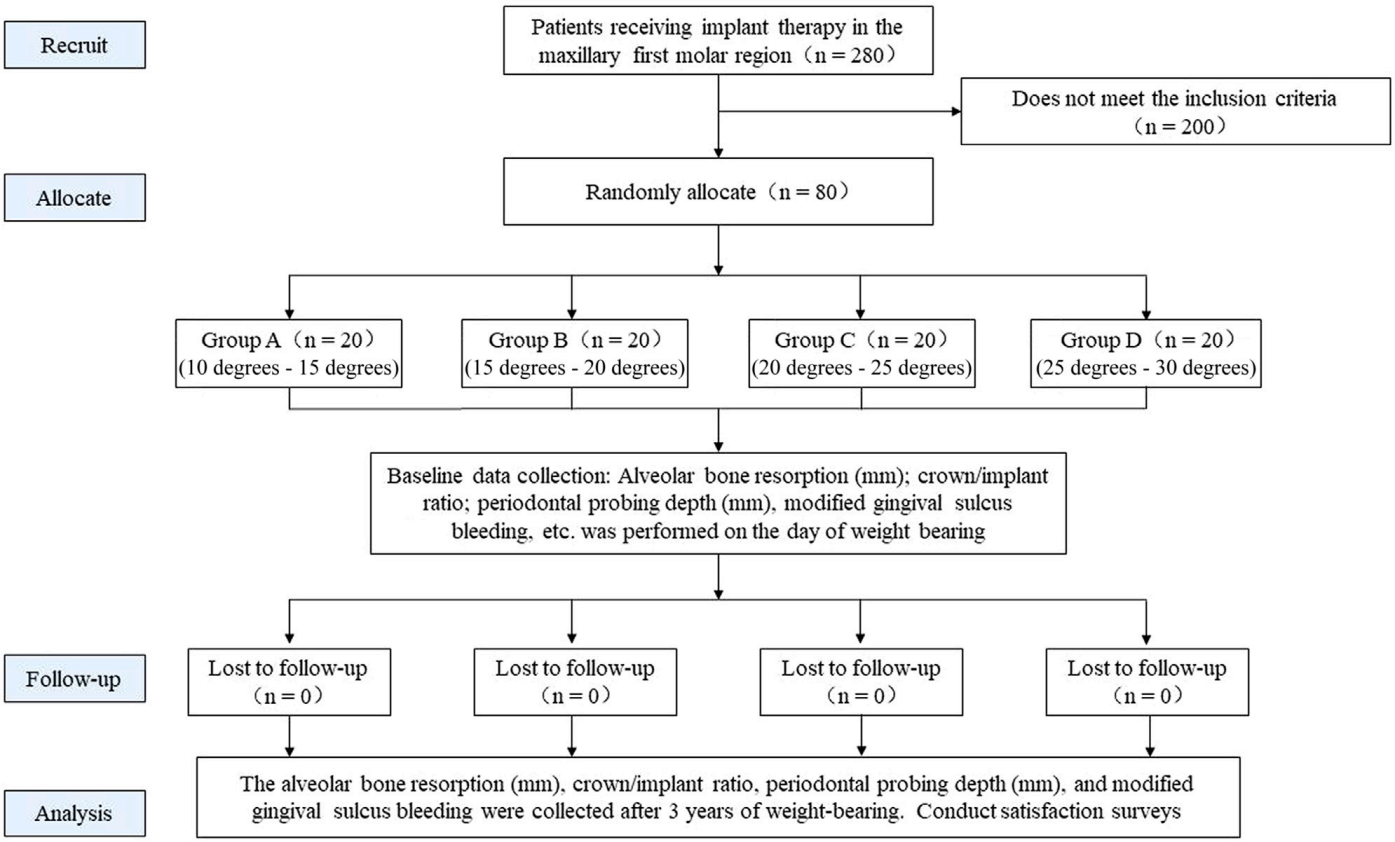
Flowchart of this study.

This study was a randomized controlled trial study. Patients who met the inclusion criteria were randomly divided into 4 groups using random numbering. The implant cusps of the four groups of patients were designed with different inclinations. After three years of follow-up, the outcome indicators of each group were observed and compared, including the amount of alveolar bone resorption in the neck of the implant restoration, periodontal probing, patients’ subjective satisfaction, and common mechanical complications within 3 years of implant restoration weight bearing. The objective of this study was to evaluate the effect of different cusp inclinations on short-implant restorations after prolonged load-bearing, and to explore the optimal cusp inclination for short-implant restorations (Figure1).

### 2.2 Study population

From August 2015 to August 2018, 280 patients, who received implant therapy in the maxillary first molar area in the Implant Center of Nantong Stomatological Hospital were selected. All patients received single crown repair.

Inclusion criteria: 1) Patient with smooth neck edge flush with the surface of the alveolar bone surface after implant placement. 2) Patients with good integration of the implants with the alveolar bone. 3) Patients with the same abutment type. 4) Patients without bad occlusal habits, such as night grinding, unilateral chewing, etc. 5) The weight-bearing of implant restorations has reached more than 3 years. 6) Patients with a stable occlusal relationship after restoration. 7) Patients with balanced biting force on bilateral teeth during occlusion. 8) Patients with coordination between retruded and centric position. 9) Patients with a large range of movement from the retruded contact position to the intercuspal position. 10) The crown/implant ratio (C/I) was controlled between 2.0 and 2.5, excluding implant restorations that were too high or too low in the sample. Exclusion criteria: 1) Patients with soft tissue lesions and periodontal disease in the missing tooth area. 2) Patients with peri-implantitis. 3) Those who have poor oral hygiene and cannot maintain it after hygiene education. This study was approved by the ethics committee of our hospital (ethics number: PJ 2015–001–01), and all patients signed informed consent. According to the results of pre-experiments, we obtained a standard deviation of the amount of alveolar bone resorption in the neck of implant restorations for each experimental group ≤ 0.3. Setting up α = 0.05, and power = 0.9, the sample size calculated using the PASS 11 software was 5.6. Therefore, 80 samples were randomly divided into 4 groups (*n* = 20).

### 2.3 Main materials and equipment

Dentium implant system (Korea, Dentium); Osstell ISQ dynamic measuring instrument (Sweden OSSTELLAB); 3 shape dental system crown restoration design software (Denmark, 3 shape company); Kavo sitting CBCT machine (German Kavo company); Florida Periodontal Probe (United States, Florida Probe Corporation).

### 2.4 Grouping method

After implant placement, observe whether the initial stability is good. A healing period of 3–6 months is required to ensure a good osseointegration between the implant and the alveolar bone. The implant stability quotient (ISQ) was measured above 75.3 shape dental system crown restoration design software (Denmark) was used to design the corresponding cusp inclination. Patients were divided into a total of 4 groups, A, B, C, and D, according to the cusp inclination. 20 cases in group A, the cusp inclination ranged from 10 degrees to 15 degrees; 20 cases in group B, the cusp inclination ranged from 15 degrees to 20 degrees; 20 cases in group C, the cusp inclination ranged from 20 degrees to 25 degrees; group D In 20 cases, the cusp inclination ranged from 25 degrees to 30 degrees. The occlusal design refers to the contralateral tooth with the same name, and the occlusion leaves a gap of 30 μm. The cusp-fossa relationship of the implant denture should be designed with a rounded and blunt arc surface contact, and an all-ceramic crown should be used for crown restoration.

### 2.5 Measurement of cusp inclination

The occlusal surface areas selected for this study were the proximal mesiolingual cusp and the central concavity. The data from the optical scanner scan (Denmark, 3 Shape) before the implant restoration was used in the DentalSystem (Denmark, 3 Shape) software to design the cusp inclination of the implant restoration based on the alignment of the teeth. Measurement method of cusp inclination: The angle between the occlusal plane and the line, which passing through both the highest point of the proximal mesiolingual cusp and the lowest point of the central concavity of the dentition, represents the cusp inclination of the proximal mesiolingual cusp. The above data were analyzed in DentalSystem (Denmark, 3 Shape) software by multiple selection of 3D models. Each index was measured 3 times and averaged as the final data.

### 2.6 Observation indicators and evaluation criteria

#### 2.6.1 Primary outcome

1) The amount of alveolar bone resorption in the neck of the implant restoration: 3 years after the implant restoration, cone beam computed tomography (CBCT) was taken. The distance from the lowest point of the alveolar bone resorption area of the implant neck to the smooth neck platform of the implant was measured at six positions near the labial and palatal sides of the implant, and the average value of the measurement results at the six positions was taken.

2) Periodontal probing: Three years after implant restoration, the Florida periodontal probe was used to probe the implant to evaluate the periodontal condition of the implant. The same experienced periodontist used the Florida periodontal probe for periodontal probing of the implant restoration, and the vertical distance from the bottom of the pocket to the gingival margin was measured, which was the probing depth (PD). Each tooth was measured at six points: proximal buccal, buccal surface, distal buccal, proximal tongue, lingual surface and distal tongue. The PD value is automatically recorded by the computer, and the value is accurate to 0.2 mm. The bleeding on probing of implant restorations was recorded according to the Modified Bleeding Index (MBI) recording standard.

3) Evaluation of common mechanical complications of implant restorations within 3 years of weight-bearing: the evaluation standard is based on the patient’s subjective feeling and doctor’s examination.

#### 2.6.2 Secondary outcome

1) The crown/implant ratio C/I of the implant restoration: measure the height (C) and implant length (I) of the crown to the abutment neck through the gingival area before wearing the tooth. The crown/implant ratio of the implant restoration was denoted as C/I.

2) Subjective satisfaction survey of patients: VAS (visual analogue scale) method (Schropp et al., 2004) was used to score the 14 questions in the questionnaire, including: 1) masticatory function; 2) and their own teeth ratio, whether you dare to bite with dental implants; 3) feel whether dental implants have become a part of your body; 4) pronunciation; 5) aesthetics; 6) whether it is convenient to clean; 7) Compared with one’s own teeth, whether dental implants are easier to clean; 8) The comfort level; 9) Do you find it troublesome to come to the hospital for follow-up or repair; 10) Whether the desired effect is achieved; 11) Whether you will still choose dental implants; 12) Whether you will recommend others for dental implants; 13) Whether it is worth the money; 14) The overall satisfaction of dental implants. The total score for each item is 100 points.

### 2.7 Statistical analysis

SPSS 22.0 software (version 25) was used for statistical analysis, the measurement data conforming to the normal distribution were expressed as mean ± standard deviation (± SD), and the comparison between groups was conducted by one-way analysis of variance; Measurement data that did not conform to the normal distribution were expressed by the median and interquartile range M (Q1, Q3), and the Wilcoxon rank sum test was used for comparison between groups. Enumeration data were expressed as n (%), and chi-square test and Fisher’s exact test were used for comparison between groups. *p* < 0.05 indicates a statistically significant difference.

## 3 Results

### 3.1 Baseline data

A total of 80 patients were included in this study, including 41 males and 39 females, with an average age of 38 ± 10 years. A total of 80 Dentium implants with a length of 8 mm and a diameter of 4.8 mm were placed in all patients. There were no significant differences in age, sex composition ratio, cervical alveolar bone resorption, crown/implant ratio, periodontal probing depth, and gingival sulcus bleeding between each group at baseline after implant restoration, indicating that the sample selection was scientific and comparable between the groups (Table1).

**TABLE 1:** Baseline characteristics of patients in each group after implant restoration.

Group	A	B	C	D
Age	39.58 ± 10.91^a^	40.15 ± 7.88^a^	35.19 ± 9.37^a^	37.2 ± 11.42^a^
Gender (male/female)	12/8^a^	11/9^a^	8/12^a^	10/10^a^
Cervical alveolar bone resorption compared with implant surgery (mm)	0.56 ± 0.23^a^	0.58 ± 0.33^a^	0.60 ± 0.29^a^	0.58 ± 0.27^a^
periodontal probing depth (mm)	1.34 ± 0.11^a^	1.28 ± 0.13^a^	1.30 ± 0.15^a^	1.29 ± 0.17^a^
Improve gingival sulcus bleeding	0.48 ± 0.21^a^	0.45 ± 0.27^a^	0.44 ± 0.19^a^	0.49 ± 0.32^a^

Note: For those with the same letter a, the difference is not statistically significant (*p* > 0.05); otherwise, the difference is statistically significant (*p* < 0.05).

### 3.2 C/I statistics of implant restorations

After implant restoration, the C/I of groups A, B, C, and D were 2.09 ± 0.28, 2.21 ± 0.32, 2.28 ± 0.21, and 2.33 ± 0.35, respectively. Statistical analysis showed that there was no significant difference between the groups, and the difference was not statistically significant. (*p* > 0.05). The results are shown in Table 2.

**TABLE 2:** Statistics of C/I after implant restoration in groups A–D.

Group	A	B	C	D
C/I	2.09 ± 0.28^a^	2.21 ± 0.32^a^	2.28 ± 0.21^a^	2.33 ± 0.35^a^

Note: For those with the same letter a, the difference is not statistically significant (*p* > 0.05); otherwise, the difference is statistically significant (*p* < 0.05).

### 3.3 The amount of alveolar bone resorption in the neck of implant restorations in each group

The amount of alveolar bone resorption in the neck of the implant restorations in each group is presented in Table 3. After implants were loaded for 3 years, the H values of teeth in groups A, B, and C were 0.71 ± 0.28, 0.74 ± 0.26, 0.78 ± 0.23, respectively, and there was no significant difference between the three groups (*p* > 0.05); The H value of group D was 1.09 ± 0.23, which was higher than that of groups A, B, and C, and the difference was statistically significant (*p* < 0.05).

**TABLE 3:** Statistics of H, PD, MBI and VAS scores of four groups A–D after 3 years of weight bearing.

Group	H	PD	MBI	VAS
A	0.71 ± 0.28^a^	1.78 ± 0.11^a^	0.69 ± 0.21^a^	82.13 ± 4.61^a^
B	0.74 ± 0.26^a^	1.85 ± 0.13^a^	0.71 ± 0.22^a^	88.36 ± 5.12^b^
C	0.78 ± 0.23^a^	1.89 ± 0.12^a^	0.71 ± 0.22^a^	88.70 ± 4.52^b^
D	1.09 ± 0.23^b^	2.19 ± 0.11^b^	0.71 ± 0.22^a^	81.86 ± 5.18^a^

a: Compared with the same letter a, there is no significant difference (*p* > 0.05); Compared with the letter b, the difference is significant (*p* < 0.05). b: Compared with the same letter b, there is no significant difference (*p* > 0.05); Compared with the letter a, the difference is significant (*p* < 0.05).

### 3.4 Periodontal probing test results

After the implants were loaded for 3 years, the PD of group D was 2.19 ± 0.11, which was significantly higher than that of groups A, B, and C, and the difference was statistically significant (*p* < 0.05); The MBI values of groups A, B, C, and D were not significantly different between groups, and the difference was not statistically significant (*p* > 0.05) (see Table 3).

### 3.5 Subjective satisfaction of patients

Subjective satisfaction of patients was reflected by the visual analogue scale VAS value (Tables 3, 4). After implants were loaded for 3 years, the VAS scores of groups B and C were 88.36 ± 5.12 and 88.70 ± 4.52, respectively, which were higher than those of groups A and D, and the difference was statistically significant (*p* < 0.05).

**TABLE 4:** Statistics of the VAS scores of four groups A–D after 3 years of weight bearing.

Question	A	B	C	D
Masticatory function	81.34 ± 4.92^a^	85.59 ± 3.93^b^	86.6 ± 5.92^b^	85.67 ± 4.72^b^
Compared with their own teeth, whether they dare to bite with implant denture	79.03 ± 9.35^a^	77.05 ± 6.32^a^	74.05 ± 5.31^a^	76.66 ± 8.35^a^
Feel whether the implant denture has become a part of their own body	89.06 ± 8.76^a^	88.06 ± 5.15^a^	90.03 ± 6.35^a^	92.05 ± 7.37^a^
Pronunciation	94.04 ± 4.94^a^	93.03 ± 5.83^a^	92.05 ± 6.94^a^	93.06 ± 5.94^a^
Aesthetic	91.53 ± 7.43^a^	92.55 ± 5.45^a^	93.37 ± 3.49^a^	93.57 ± 5.47^a^
Whether cleaning is convenient	80.51 ± 5.92^a^	83.52 ± 6.14^a^	86.55 ± 7.54^a^	88.58 ± 8.82^a^
Compared with their own teeth, whether implant denture is easier to clean	73.87 ± 5.93^a^	75.67 ± 3.73^a^	78.48 ± 8.03^a^	77.34 ± 7.94^a^
Degree of comfort	94.53 ± 3.47^a^	93.58 ± 3.63^a^	91.52 ± 4.63^a^	92.93 ± 4.56^a^
Whether it is troublesome to come to the hospital for further consultation or repair	73.38 ± 3.84^a^	73.33 ± 3.53^a^	73.35 ± 6.17^a^	73.36 ± 7.93^a^
Whether the expected effect is achieved	83.23 ± 5.85^a^	90.25 ± 5.13^b^	90.28 ± 4.34^b^	80.26 ± 4.15^a^
whether implant denture will still be selected	95.03 ± 3.76^a^	92.06 ± 6.55^a^	94.04 ± 5.86^a^	96.03 ± 3.36^a^
whether they will recommend implant denture to others	93.06 ± 5.54^a^	92.03 ± 4.46^a^	92.03 ± 6.24^a^	90.04 ± 6.52^a^
whether it is worth the money	85.35 ± 9.28^a^	88.39 ± 7.53^a^	88.35 ± 4.22^a^	88.38 ± 8.83^a^
Overall satisfaction of implant denture	82.13 ± 4.62^a^	88.36 ± 5.12^b^	88.70 ± 4.52^b^	81.86 ± 5.18^a^

Within the same column, the values (mean ± SD) with asterisk are significantly different (one-way ANOVA, analysis and SNK-q, test, *p* < 0.05).

### 3.6 Statistics of common mechanical complications of implant restorations within 3 years of weight bearing

The common mechanical complications of the four groups of patients within 3 years of implant restoration were food impaction, porcelain collapse, and abutment loosening. The statistics are shown in Table 5. The probability of implant collapse and abutment loosening in group D within 3 years of weight-bearing were 25.0% (5/20) and 15.0% (3/20), which were significantly higher than those in groups A, B, and C (*p* < 0.05).

**TABLE 5:** Statistics of common mechanical complications within 3 years of weight-bearing in groups A–D.

Group	Food impaction (%)	Broken porcelain (%)	Abutment loose (%)
A	50.0% (10/20)	15.0% (3/20)^*^	0.0% (0/20)^*^
B	25.0% (5/20)^*^	10.0% (2/20)^*^	5.0% (1/20)^*^
C	30.0% (6/20)^*^	15.0% (3/20)^*^	5.0% (1/20)^*^
D	40.0% (8/20)	25.0% (5/20)	15.0% (3/20)

Note: Compared with group D, no * in groups A, B and C means no significant difference (*p* > 0.05); * means significant difference (*p* < 0.05).

### 3.7 Implant survival rate after 3 years of implant restoration

The implant retention rates of the four groups A to D were all 100% after 3 years of implant restoration.

## 4 Discussion

Short implant prostheses overcome the problem of more invasive procedures due to insufficient bone mass and are being accepted by more clinicians. At present, studies have shown that there was no significant difference in the survival rate of short implants and standard length implants after three years (Jung et al., 2018; Papaspyridakos et al., 2018). Factors such as implant length, implant shape, and implantation technique have a limited impact on the long-term clinical success of short implants. In this study, the implant retention rates of the four groups of samples after three years of occlusal load-bearing were all 100%, indicating that the cusp inclination had no significant effect on the short-term retention rate of short implant restorations. However, biomechanical studies have shown that higher C/I (crown/implant ratio) may increase the stress of the bone tissue around the implant neck and lead to marginal bone loss (Sotto-Maior et al., 2015). A prospective clinical study also confirmed that higher C/I lead to more bone resorption at the implant margin (Pierrisnard et al., 2010). In this study, we excluded patients with high or low C/I values to reduce the influence of restoration C/I value on cusp inclination, a single factor study, and to control for potential confounding factors.

The research on the influence of cusp inclination on the hard tissue of the implant neck is currently more based on three-dimensional finite element analysis. Most of the research results believe that when the cusp inclination is greater than 25 degrees, the stress on the alveolar bone of the implant neck increases with the increase of the cusp inclination (Zhou et al., 2003; Han and Li, 2004). In this study, it was also found that when the cusp inclination was greater than 25 degrees, the amount of marginal bone resorption at the neck of the short implant increased, and the difference was statistically significant (*p* < 0.05). However, some 3D finite element analysis experiments concluded that the effect of cusp inclination on the hard tissue of the implant neck was not significant (Rosse et al., 2010; Sodnom-Ish et al., 2020). The study of Zhou et al. (2003) replaced a more reasonable loading method, and the results showed that with the increase of the cusp inclination, the stress value of the cortical bone of the implant neck increased significantly. Therefore, differences in conclusions between different studies may be caused by different loading methods.

Some studies have confirmed that a reasonable occlusal design can help maintain the long-term health of the soft and hard tissues around the implant and prolong the service life of the denture (Graves et al., 2016). To maintain the long-term integrity of peri-implant osseointegration, it is necessary to maintain a dynamic balance between occlusal force-induced microtrauma and self-healing capacity. If the rate of microtrauma exceeds the self-healing capacity, it will exhibit bone resorption around the neck of the implant (Brunski, 1992). McCullock believes that the occlusal force transmitted to the implant restoration should be controlled within the physiological limit that the patient’s oral and maxillary system can withstand, so as to avoid bone tissue resorption around the implant restoration and maintain the health of the gingival tissue around the implant restoration (McCullock, 2003). In this study, strict restrictions were placed on the occlusal design of each implant restoration. Due to its relatively fixed position, the maxilla has a lower ability to avoid buffering than the mandible, and the maxillary first molars are subjected to greater passive impact when they perform chewing function (Jiang and Li, 2003). Therefore, in this study, patients who received implant treatment of the maxillary first molar receiving area were selected as the research subjects.

Before 2018, the international consensus for short implant restorations was ≤ 8 mm in length, whereas after 2018 the consensus for short implants was ≤ 6 mm. Most domestic clinicians use the implant length ≥ 7.5 mm in the maxillary first premolar region. In this study, a short implant of 8 mm was selected for clinical research. The change in bone height at the implant edge is an important indicator to measure the success of the implant, and the evaluation standard is bone resorption < 1 mm in the first year after repair and < 0.2 mm in subsequent years. In this study, no cases of peri-implant alveolar bone resorption > 1.4 mm were found after 3 years of implant restoration. Wu et al. (2010) confirmed that the marginal bone resorption of short implants after 3 years of load-bearing was 0.69 mm–0.86 mm. Some scholars conducted a retrospective study of 247 short and ultra-short implants with weight bearing for 3 years, and the results showed that the average alveolar bone resorption was 0.6 mm–0.7 mm (Draenert et al., 2012). This result is similar to that of groups A to C in this study. Vazouras et al. found that the majority of short implant failures (≤ 6 mm) occurred 3 years after loading (Papaspyridakos et al., 2018). In this study, the survival rate of implants after 3 years of implant loading was 100%, but when the cusp inclination exceeds 25 degrees, the long-term preservation rate of implant restorations deserves further follow-up.

The increase in PD in group D in this study may be directly caused by increased alveolar bone resorption. However, the correlation between alveolar bone resorption and the periodontal depth of natural teeth or non-implant dentures does not indicate that alveolar bone resorption will inevitably lead to an increase in PD. Studies have confirmed that when alveolar bone resorption is about 0.75 mm, the attachment of natural teeth is lost by about 0.65 mm (Chen et al., 2017), and the correlation of PD increases increases. In this study, there were 8 (40%) implants with 1 mm of alveolar bone resorption in group D, which may be an important reason for the increase in PD. However, group D did not show a significant increase in MBI. Studies have confirmed that when PD < 2.37 mm, periodontal or gingival inflammation is not obvious, and when PD > 2.37 mm, the risk of periodontal disease or peri-implant inflammation increases (Chen et al., 2017). The four groups of implants in this study had PD < 2.37 mm, which is an important reason why the MBI in group D did not increase significantly after 3 years of implant restoration.

Zhang Yinan in the study of cusp inclination on conventional implants, believed that the overall satisfaction of patients was higher when the cusp inclination was 15 degrees–25 degrees, and some scholars believed that the cusp inclination was less than 25 degrees when implanted with an all-ceramic crown. more appropriate (Zhang et al., 2009). In this study, the results of the patient satisfaction survey showed that the patient’s satisfaction was higher when the cusp inclination was between 15 degrees and 25 degrees. Among them, there was a high correlation between masticatory function and overall satisfaction, suggesting that patients with cusp inclination of 15 degrees–25 degrees had higher masticatory efficiency and better expected results. The adjustment of occlusal freedom is an important method to avoid abnormal occlusal force and prevent occlusal trauma. The larger the cusp inclination angle, the smaller the occlusal degree of freedom in the middle occlusal position, which restricts the movement of the mandible (Fang et al., 2019). Patient satisfaction scores in the group with cusp inclination > 25 degrees in this study were lower than those in the group with cusp inclination between 15 degrees and 25 degrees. In addition, the masticatory function of patients was also limited when the cusp inclination was too low, so this may be the main reason for the lower patient satisfaction score in group A in this study.

In this study, it was found that the probability of food impaction of implant restorations in patients with cusp angle > 25 degrees was higher than that in patients with cusp angle < 25 degrees. This is because the component force generated by the implant restoration with high cusp inclination during the chewing process pushes the natural teeth to move in the opposite direction, the friction frequency of adjacent teeth increases, and the probability of long-term wear of the adjacent surface increases (Incau et al., 2012; Günay et al., 2000). The incidence of food impaction was also higher in the 10 degrees-15 degrees group in this study. After careful investigation of the cases, it was found that these patients often have tooth wear and the downward movement of the adjacent zone. Normal abutment disruption should be the primary cause of food impaction. There are many investigations and studies on implant restorations, and most of the studies have confirmed that the greater the cusp inclination, the higher the porcelain chipping rate, so this is the reason for the high porcelain chipping rate in the 25 degrees–30 degrees group in this study. Abutment loosening is often mentioned in the mechanical complications of implant restoration. In this study, the incidence of abutment loosening in the 25 degrees–30 degrees group was higher than that in the other three groups. However, this complication mostly occurred after implant restoration. In the early stage, it is mostly related to the incomplete seating of the abutment. In the 25 degrees–30 degrees group, it was found that a larger proportion of adjacent teeth were inclined to the missing area, which may be the main reason for the incomplete seating of screw-retained implant restorations.

This study has certain limitations. First, this study is a single-center randomized controlled trial study with a small sample size. Further multi-center studies with a large sample size are needed to verify the conclusions of this study. Second, this study could not rule out potential confounding factors such as the patient’s diet, dental hygiene habits, etc., which may have some influence on the final results. Third, there is a lack of statistical analysis of data from intermediate nodes such as implants after 1/2 years. Fourth, the implant length chosen for our study was 8 mm, which may not show the advantage of a real “short implant”. We need to investigate the effect of shorter implant lengths. Fifth, only patients with maxillary dental restorations were included in our study. Although the original study design was designed to control for confounding factors and to make the variables uniform, this limits the generalization of the findings of this study, because in clinical practice, patients with mandibular restorations should also be considered. Moreover, the crown/implant ratio of the patients included in this study was controlled between 2.0 and 2.5, which differs from the real clinical situation. In future studies, we will include all patients with different crown/implant ratios and design more rigorous studies to explore more valuable results. Future research in other cohorts will be needed to verify our findings.

## 5 Conclusion

Short-term implant restorations with high cusp inclination may increase the risk of biological and mechanical complications after long-term load bearing. The cusp inclination of short-implant restorations should be designed to be < 25 degrees as far as possible. Longer clinical follow-up and observation are needed for the effect of cusp inclination on the survival rate of short implants.

## Data Availability

The original contributions presented in the study are included in the article/supplementary material, further inquiries can be directed to the corresponding author.

## References

[B1] ’Incau E. D. Couture C. Maureille B. (2012). Human tooth wear in the past and the present: Tribological mechanisms, scoring systems, dental and skeletal compensations. Arch. Oral Biol. 57 (3), 214–229. 10.1016/j.archoralbio.2011.08.021 21920497

[B2] Altaib F. H. Alqutaibi A. Y. Al-Fahd A. Eid S. (2019). Short dental implant as alternative to long implant with bone augmentation of the atrophic posterior ridge: A systematic review and meta-analysis of RCTs. Quintessence Int. 50 (8), 636–650. 10.3290/j.qi.a42948 31372602

[B3] Bechara S. Kubilius R. Veronesi G. Pires J. T. Shibli J. A. Mangano F. G. (2017). Short (6-mm) dental implants versus sinus floor elevation and placement of longer (≥10-mm) dental implants: A randomized controlled trial with a 3-year follow-up. Clin. Oral Implants Res. 28 (9), 1097–1107. 10.1111/clr.12923 27402427

[B4] Brocard D. Barthet P. Baysse E. Duffort J. F. Eller P. Justumus P. (2000). A multicenter report on 1, 022 consecutively placed ITI implants: A 7-year longitudinal study. Int. J. Oral Maxillofac. Implants 15 (5), 691 11055136

[B5] Brune A. Stiesch M. Eisenburger M. Greuling A. (2019). The effect of different occlusal contact situations on peri-implant bone stress - a contact finite element analysis of indirect axial loading Mat. Sci. Eng. C Mat. Biol. Appl. 99, 367–373. 10.1016/j.msec.2019.01.104 30889710

[B6] Brunski J. B . (1992). Biomechanical factors affecting the bone-dental implant interface. Clin. Mat. 10 (3), 153–201. 10.1016/0267-6605(92)90049-y 10149982

[B7] Chen Q. Zhou Z. Zhou Y. (2017). [A study on effects of immediate bone grafting at mandibular first molar fresh extraction socket on maintaining alveolar bone height after space closure]. Chin. J. Stomatol. 52 (11), 649–655. 10.3760/cma.j.issn.1002-0098.2017.11.001 29972942

[B9] Cutolo M. A. Cafiero C. Califano L. Giaquinto M. Cusano A. Cutolo A. (2022). Feasibility analysis of an ultrasound on line diagnostic approach for oral and bone surgery. Sci. Rep. 12 (1), 905. 10.1038/s41598-022-04857-0 35042892 PMC8766520

[B10] Draenert F. G. Sagheb K. Baumgardt K. Kammerer P. W. (2012). Retrospective analysis of survival rates and marginal bone loss on short implants in the mandible. Clin. Oral Implants Res. 23 (9), 1063–1069. 10.1111/j.1600-0501.2011.02266.x 22092574

[B11] Fang C. Y. Yu J. H. Chang C. C. Hsu J. T. Lee Y. C. Huang H. L. (2019). Effects of short-term acupuncture treatment on occlusal force and mandibular movement in patients with deep-bite malocclusion. J. Dent. Sci. 14 (1), 81–86. 10.1016/j.jds.2018.11.003 30988883 PMC6445974

[B12] Felice P. Barausse C. Pistilli R. Ippolito D. R. Esposito M. (2019). Five-year results from a randomised controlled trial comparing prostheses supported by 5-mm long implants or by longer implants in augmented bone in posterior atrophic edentulous jaws. Int. J. Oral Implantol. 12 (1), 25 31116186

[B13] Glücker C. Rauch A. Hahnel S. (2020). Attitude and treatment options in implant-supported prosthetics: A survey among a cohort of German dentists. J. Adv. Prosthodont. 12 (1), 15–21. 10.4047/jap.2020.12.1.15 32128082 PMC7040449

[B14] Graves C. V. Harrel S. K. Rossmann J. A. Kerns D. Gonzalez J. A. Kontogiorgos E. D. (2016). The role of occlusion in the dental implant and peri-implant condition: A review. Open Dent. J. 10 (1), 594–601. 10.2174/1874210601610010594 27990184 PMC5123128

[B15] Günay H. Seeger A. Tschernitschek H. (2000). Placement of the preparation line and periodontal health--a prospective 2-year clinical study. Int. J. Periodontics Restor. Dent. 20 (2), 171 11203559

[B16] Han D. Li Q. (2004). Effects of different cusp inclinations on the stress distribution of a single implant denture. J. Pract. Stomatology. 10.3969/j.issn.1001-3733.2004.02.008

[B17] Jiang L. Li W. (2003). Clinical observation and etiological analysis of cracked teeth. J. Stomatol. 23 (2), 2. 10.3969/j.issn.1003-9872.2003.02.013

[B18] Jung R. E. Al-Nawas B. Araujo M. Avila-Ortiz G. Barter S. Brodala N. (2018). Group 1 ITI Consensus Report: The influence of implant length and design and medications on clinical and patient‐reported outcomes Clin. Oral Implants Res. 29, 69–77. 10.1111/clr.13342 30328189

[B19] Kim J. H. Lim Y. J. Kim B. Lee J. (2021). How do parameters of implant primary stability correspond with CT-evaluated bone quality in the posterior maxilla? Materials 14 (2), E270. 10.3390/ma14020270 PMC7828085 33430383

[B20] Kim Y. Oh T. J. Misch C. E. Wang H. L. (2005). Occlusal considerations in implant therapy: Clinical guidelines with biomechanical rationale. Clin. Oral Implants Res. 16 (1), 26–35. 10.1111/j.1600-0501.2004.01067.x 15642028

[B21] Manchikalapudi G. Basapogu S. (2022). Finite Element Analysis of effect of cusp inclination and occlusal contacts in PFM and PEEK implant-supported crowns on resultant stresses. Med. J. Armed Forces India 78 (1), 80–87. 10.1016/j.mjafi.2020.11.014 35035048 PMC8737102

[B22] McCullock A. J . (2003). Making occlusion work: I. Terminology, occlusal assessment and recording. Dent. Update 30 (3), 150–157. 10.12968/denu.2003.30.3.150 12743913

[B23] Misch C. E. Steigenga J. Barboza E. Misch-Dietsh F. Cianciola L. J. Kazor C. (2006). Short dental implants in posterior partial edentulism: A multicenter retrospective 6-year case series study. J. Periodontol. 77 (8), 1340–1347. 10.1902/jop.2006.050402 16937587

[B24] Monje A. Suarez F. Galindo-Moreno P. Garcia-Nogales A. Fu J. H. Wang H. L. (2014). A systematic review on marginal bone loss around short dental implants (<10 mm) for implant-supported fixed prostheses. Clin. Oral Implants Res. 25 (10), 1119–1124. 10.1111/clr.12236 23937287

[B25] Papaspyridakos A. Vazouras K. Gholami H. Pagni S. Weber H. P. (2018). Survival rates of short dental implants (≤6 mm) compared with implants longer than 6 mm in posterior jaw areas: A meta-analysis. Clin. Oral Implants Res. 29 (16), 8–20. 10.1111/clr.13289 30328206

[B26] Pierrisnard L. Renouard F. Renault P. Barquins M. (2010). Influence of implant length and bicortical anchorage on implant stress distribution Clin. Implant Dent. Relat. Res. 5 (4), 254–262. 10.1111/j.1708-8208.2003.tb00208.x 15127996

[B27] Rosse Mary Falcón-Antenucci De Carvalho P. S. P. Goiato M. C. Noritomi P. Y. (2010). Influence of cusp inclination on stress distribution in implant-supported prostheses. A three-dimensional finite element analysis. J. Prosthodont. 19 (5), 381–386. 10.1111/j.1532-849x.2010.00582.x 20345742

[B28] Schropp L. Isidor F. Kostopoulos L. Wenzel A. (2004). Patient experience of, and satisfaction with, delayed-immediate vs. delayed single-tooth implant placement. Clin. Oral Implants Res. 15 (4), 498–503. 10.1111/j.1600-0501.2004.01033.x 15248886

[B29] Sodnom-Ish B. Eo M. Y. Nguyen T. T. H. Kim M. J. Kim S. M. (2020). Clinical feasibility and benefits of a tapered, sand-blasted, and acid-etched surfaced tissue-level dental implant. Int. J. Implant Dent. 6 (1), 39. 10.1186/s40729-020-00234-6 32761304 PMC7406589

[B30] Sotto-Maior B. S. Senna P. M. Silva-Neto J. de Arruda Nobilo M. A. Del Bel Cury A. A. (2015). Influence of crown-to-implant ratio on stress around single short-wide implants: A photoelastic stress analysis. J. Prosthodont. 24 (1), 52–56. 10.1111/jopr.12171 24919655

[B31] Stellingsma K. Raghoebar G. M. Meijer H. Stegenga B. (2004). The extremely resorbed mandible: A comparative prospective study of 2-year results with 3 treatment strategies. Int. J. Oral Maxillofac. Implants 19 (4), 563 15346755

[B32] Thoma D. S. Cha J. K. Jung U. W. (2017). Treatment concepts for the posterior maxilla and mandible: Short implants versus long implants in augmented bone. J. Periodontal Implant Sci. 47 (1), 2–12. 10.5051/jpis.2017.47.1.2 28261519 PMC5332331

[B33] Wu H. Li J. Qiu L. x. Lin Y. Luo J. (2010). [A long-term retrospective clinical study of short dental implant restoration]. Chin. J. Stomatology 45 (12), 712. 10.3760/cma.j.issn.1002-0098.2010.12.003 21211234

[B34] Zhou H. Sun Y. Zhu B. (2003). Three-dimensional finite element analysis of the design of different cusp inclinations for implant restoration of upper dentures. J. Tianjin Med. Univ. 9 (2), 4. 10.3969/j.issn.1006-8147.2003.02.038

[B8] Zhou Y. (2019). Study on the influence of two groups of different cusp inclination bite designs on the peri-implant tissue. J. China Medical Device Information (2), 13. 10.3969/j.issn.1006-6586.2019.13.069

